# Single molecule super-resolution imaging of bacterial cell pole proteins with high-throughput quantitative analysis pipeline

**DOI:** 10.1038/s41598-019-43051-7

**Published:** 2019-04-30

**Authors:** Ipek Altinoglu, Christien J. Merrifield, Yoshiharu Yamaichi

**Affiliations:** 1 Department of Genome Biology, Institute for Integrative Biology of the Cell (I2BC), Université Paris-Saclay, CEA, CNRS, Univ. Paris Sud, Gif sur Yvette, France; 2 Department of Cell Biology, Institute for Integrative Biology of the Cell (I2BC), Université Paris-Saclay, CEA, CNRS, Univ. Paris Sud, Gif sur Yvette, France; 3 Graduate School of Structure and Dynamics of Living Systems, Univ. Paris-Sud, Orsay, France

**Keywords:** Super-resolution microscopy, Bacterial techniques and applications

## Abstract

Bacteria show sophisticated control of their cellular organization, and many bacteria deploy different polar landmark proteins to organize the cell pole. Super-resolution microscopy, such as Photo-Activated Localization Microscopy (PALM), provides the nanoscale localization of molecules and is crucial for better understanding of organization and dynamics in single-molecule. However, analytical tools are not fully available yet, in particular for bacterial cell biology. For example, quantitative and statistical analyses of subcellular localization with multiple cells from multiple fields of view are lacking. Furthermore, brightfield images are not sufficient to get accurate contours of small and low contrast bacterial cells, compared to subpixel presentation of target molecules. Here we describe a novel analytic tool for PALM which integrates precisely drawn cell outlines, of either inner membrane or periplasm, labelled by PALM-compatible fluorescent protein fusions, with molecule data for >10,000 molecules from >100 cells by fitting each cell into an oval arc. In the vibrioid bacterium *Vibrio cholerae*, the polar anchor HubP constitutes a big polar complex which includes multiple proteins involved in chemotaxis and the flagellum. With this pipeline, HubP is shown to be slightly skewed towards the inner curvature side of the cell, while its interaction partners showed rather loose polar localization.

## Introduction

Bacterial cells sophisticatedly place their biological apparatuses inside and outside of the cell for their proper function. In rod-shaped bacteria, cell poles play an important role for the systematic arrangement of multi-component cellular processes such as chromosome dynamics, cell cycle, development, active transport, chemotaxis and motility^1^. The localization pattern of polar proteins varies from stable localization at one or the pole to movement from one pole to the other during the cell cycle^2^. Understanding the control mechanisms of polarity in space and time is now appreciated as a crucial question in bacterial cell biology.

Many bacterial species deploy polar landmark protein(s) to recruit certain protein complexes by protein-protein interactions thus organizing polar functions. DivIVA, originally identified in *Bacillus subtilis*, localizes at the cell pole by recognizing negative curvature^3,4^. In *B. subtilis* and other Firmicutes, DivIVA has been shown to recruit the sporulation-specific chromosome segregation protein RacA^5^, cell division inhibitor complex MinCD (through MinJ and/or via direct interaction with MinD)^6–8^, and plausibly protein(s) involved in autolysin secretion and swarming^9,10^. DivIVA homologs in Actinomyces are also shown to interact with chromosome segregation complex ParAB, polar peptidoglycan biosynthesis machinery, and an intermediate filament-like protein FilP^11–14^. Recently, DivIVA in coccoid *Staphylococcus aureus* is also shown to interact with several proteins including bacterial condensin SMC^15^.

In *Caulobacter crescentus* (and other alpha-proteobacteria), membrane-bound TipN and self-assembling cytoplasmic protein PopZ serve polar organizers of new and old cell pole, respectively. They play an important role during chromosome segregation by interacting with ParA and/or ParB^2,16^. PopZ particularly acts as ‘hub’ protein by directly interacting with more than a dozen proteins involved in various cellular processes including cell cycle regulation, development and motility^17–19^.

Recently in Gram negative *Myxococcus xanthus*, three bactofilin proteins called BacNOP have been found to co-assemble into an extended structure at the cell poles which acts as scaffold of ParAB and another small GTPase SofG involved in motility^20,21^.

Lastly in *Vibrio* and *Shewanella* species, the transmembrane protein HubP serves as a polar landmark. In *V. cholerae*, HubP was shown to tether the origin of replication of the larger chromosome 1 by interaction to ParA1, and is thus implicated in chromosome segregation as well as division site selection via nucleoid occlusion^22,23^. HubP is also involved in polar assembly of chemotaxis and flagella apparatus by recruiting proteins such as ParC (polar localization of chemotaxis apparatus), FlhG and SflA^22,24–26^. The HubP homolog in *Pseudomonas aeruginosa*, FimV, recruits both physical components (Type IV pili) and a regulator (adenylate cyclase) for the twitching motility to the cell pole^27–29^.

*V. cholerae*, the causative agent of the deadly diarrheal disease cholera, is a curved rod shaped Gram negative bacteria which is highly motile with a polar monotrichous flagellum. Chemotaxis and motility have been shown to play important roles in intestinal colonization, virulence and transmission of *V. cholerae*^30,31^. Recently, the periplasmic protein CrvA was identified to be the determinant of cell curvature by altering the insertion of peptidoglycan. Inactivation of CrvA resulted in straight cell shape and alleviated gel matrix motility and pathogenicity^32^.

Imaging bacterial cells with fluorescence microscopy has been a keystone of modern bacterial cell biology from identification and characterization of proteins to shedding light on orchestrated cellular machineries and processes including cytoskeleton, cell division and chromosome transactions^33,34^. Particularly, live cell imaging allowed the detection of protein interactions as well as dynamics, both at the single cell level and in populations of bacteria^35^. Furthermore, quantitative image analyses, from morphology of cells to detection and quantification of fluorescent foci, has become a powerful tool particularly with a systems biology approach^36^. Several pieces of software such as MicrobeTracker, Oufti, supersegger, BactImAS and MicrobeJ have been developed to facilitate these analyses^37–41^. Even though these programs use algorithms to handle subpixel resolution, light microscopy is subject to the diffraction limit, which restricts the maximum spatial resolution achievable. In the last decade, so-called ‘super-resolution’ techniques challenged this problem, such as stimulated emission depletion (STED), photoactivation localization microscopy (PALM), stochastic optical reconstruction microscopy (STORM), structured illumination microscopy (SIM) and saturated excitation (SAX)^42^.

PALM takes advantage of photo activatable-/switchable-fluorescent proteins (FPs) along with stochastic activation of isolated fluorophores. Thousands of cycles of activation-excitation-bleaching followed by computational manipulation result in imaging with a resolution of ~10–30 nm^42^. Commercially available PALM accompanied with dedicated software (for instance, N-STORM from Nikon) allows the detection and localization of single molecules for each excitation cycle and combines the localizations over thousands of images with user-defined parameters. In addition to obtaining intracellular structure at fine scale, cluster analysis has been applied in bacteria, which sheds light on dynamics and the mode of self-assembly of proteins along the cell^43^. PALM has also been successful for tracking the trajectory of single molecules for short periods of time, to elucidate the diffusive state of bacterial proteins^44,45^. Currently, however, there is a limitation for quantitative and statistical analyses of subcellular localization from multiple cells (from one or multiple fields of view). Not only does N-STORM not handle such analyses, but images consist in lists of detected molecules which is a format not compatible with established image analysis tools for fluorescence microscopy images. An automated modality of PALM, HTPALM, has been developed for high-throughput imaging and analysis^46^. However, it requires specific hardware including a custom built microscope for automation and external phase contrast microscopy^46^. Therefore, there is a demand to develop means to transfer a PALM molecule list corresponding to protein locations within a cell with high-throughput capability.

Here, we study the precise localization of polar proteins in *V. cholerae* with super-resolution PALM. To this end, we built a Matlab-based software Vibio, which combines PALM detected molecule lists with cell meshes which are drawn by MicrobeTracker. We show that using brightfield (BF) images are not sufficient for precise localization analysis. Therefore we present a novel cell outline technique in which the inner membrane or the periplasm is labelled with photo-activatable/switchable FPs. We also show that Vibio can distinguish inner and outer curvature of curved-rod cells. Altogether, we show that HubP is rather localized to the inner curvature from the tip of pole, while its interaction partners have distinct localization patterns. This new labelling method and localization software will provide a better landscape of localization for single molecules in populations of cells.

## Results

### Different polar clusters of HubP by expression level

**Figure 1:**
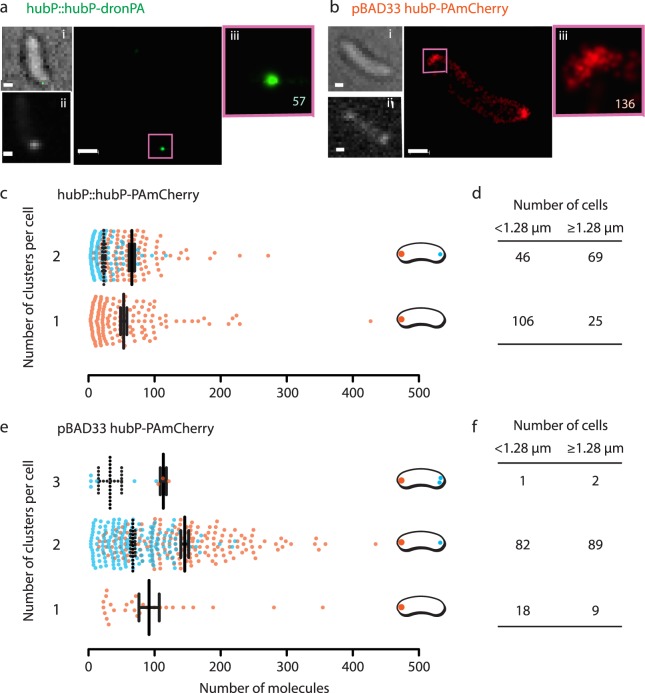
Polar HubP clusters. (**a,b**) Representative image of cell with native level (**a**) or overexpressed (**b**) HubP-FPs. Corresponding out-of-focus BF image (**i**), conventional fluorescent image (ii) are also shown. The region in the purple square is magnified in (iii). Bar = 500 nm. (**c–f**) Distribution of HubP clusters in native level expression (c and d) or overexpressed (**e,f**) conditions. (**c,e**) Dot plots of number of molecules per cluster. For ≥2 clusters per cell, the cluster with highest number of molecules was indicated in red and other clusters were shown in blue. The mean and standard error of mean are also indicated. (**d,f**) Number of cells containing 1, 2, or 3 clusters of HubP molecules with respect to cell size. 1.28 µm is the average cell size for these experiments.

In the previous study on the polar localization of HubP, we utilized an arabinose-inducible overexpression vector system in which green, yellow, or cyan FP was fused to the cytoplasmic C-terminal end of HubP^22^. To carry out PALM, we constructed new plasmids by changing the fluorophore to PALM-compatible DronPA and PAmCherry. We also replaced chromosomal *hubP* by *hubP-dronPA* or *hubP-PAmCherry* fusion to investigate protein localization under native expression level (Supplementary Fig. S1c). A few apparent differences were observed between cells with overexpression (~70 x at mRNA level, Supplementary Fig. S1c) and native level expression of HubP. First, in contrast to the vast majority of cells which had bipolar signals when overexpressed (which is consistent with our previous study)^22^, chromosomally-encoded HubP showed mixed populations of cells with uni- and bi-polar signal. Notably, under overexpression conditions, detected HubP molecules are often observed as ‘cap’ rather than ‘focus’ (Fig. 1a,b).

For further understanding of HubP localization from a quantitative point of view, we carried out cluster analysis with SR-Tesseler^47^. When HubP-PAmCherry was expressed from an endogenous locus, the majority of younger cells (shorter than the average cell size of 1.28 µm) had 1 cluster at one cell pole. Bipolar clusters appeared in longer cells and these cells presented significantly more molecules than cells with only 1 cluster. Notably, bipolar clusters of HubP showed a skewed pattern of number of molecules (Fig. 1c,d). Presumably, in a newborn cell, HubP clustered at the old cell pole. As the cell cycle progresses, HubP molecules accumulate into the existing cluster as well as form a new cluster at the new cell pole (discussed later).

It is no wonder that a much higher total number of HubP-PAmCherry molecules were detected in overexpressing cells. Yet, cluster analysis indicated that HubP molecules are organized into only a single cluster at each cell pole in nearly all the cases, even though they often assemble into a cap shape (Fig. 1e,f). It is possible that higher accumulation of molecules in the cluster resulted in the ‘cap’ form. Furthermore, the polarity of the cell could be maintained even in the overexpression condition, as the number of molecules in the two clusters remained skewed in these cells. Nonetheless for further investigation, we used chromosomal fusion and studied localization at native expression level.

### Incorporation of molecules into cells

**Figure 2:**
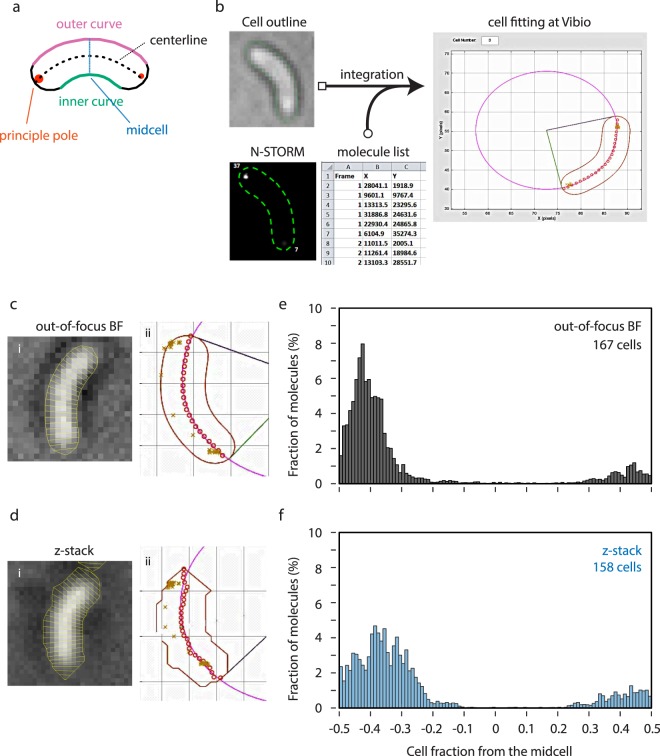
Vibio, high-throughput quantitative PALM molecule localization analysis pipeline. (**a**) Schematics of *V. cholerae* cell. The pole with more detected molecule is determined as principle pole. (**b**) Graphical abstract of Vibio pipeline. (**c**,**d**) (i) Representative cell of single out-of-focus BF image (**c**) or reconstructed image from 32 z-stacks (**d**). Outline and segmentation are shown by a yellow line. (ii) Fitting of corresponding cell in Vibio. Each detected HubP molecule is plotted with ‘x’. (**e**,**f**) Histogram presentation of the localization of HubP molecules relative to the long axis of the cell determined by indicated outlining method. −0.5 indicates the principle pole and +0.5 indicates the other pole.

In N-STORM, detected molecules can be visualized as crosses (see Supplementary Fig. S4a) or rendered to Gaussian blur. While some analyses such as cluster analysis discussed above can be handled manually, others definitely require computational processing of data. As mentioned, however, no high-throughput software supports lists of molecules in x and y coordinates. Therefore, we had to develop a custom solution to integrate PALM results into the cell, with high-throughput capability. On this account, we developed Matlab-based software with a Graphical User Interface (GUI) (named Vibio) to combine molecules (in x, y coordinates) and cell shape information. For each cell outline, the corresponding molecular positions are extracted from the list and located accordingly in the cell. To present the localization data, Vibio draws an oval in which the cell centreline fits then the arc angle is used to calculate the cell length as well as the distance between the molecules and the cell poles (Fig. 2b). We also set one pole as ‘principle pole’ which contains a higher amount of molecules compared to the opposite pole for standardized orientation. In our study of HubP localization, this principle pole corresponds to the old cell pole (Fig. 2a).

The GUI of Vibio includes display of results either as a histogram or as an oriented 2-dimentional map (see below) for each cell or for all the cells from one field of view (typically ~15 cells). The results can be also exported as an Excel file so that we can analyse > 100 cells with thousands of molecules by compiling multiple fields. Unexpectedly, the initial analysis of HubP localization (15,325 molecules from 217 cells) by Vibio showed that HubP was not restricted to cell poles but rather distributed in region of 0–20% of the cell length (Fig. 2e). With careful revision of data, it became clear that insufficient segmentation from BF image resulted in mediocre cell outlining unsuitable for proper analysis in super-resolution (see below).

### Novel outlining technique

To better visualize the cells and facilitate the detection of their outlines, fluorescence microscopy is often combined with phase contrast microscopy in which cells appear dark over a bright background. Programs widely used for the analysis of conventional fluorescence microscopy images are designed to use these phase contrast images. However, phase contrast imaging is not usually compatible with PALM or other super-resolution microscopes^46^. With BF imaging, bacterial cells at the focal plane are barely detectable and only become adequately clear with some defocus (e.g. −200 nm, Fig. 2c). Instead of using one out-of-focus BF image, the detection of cell contour from z-stack BF images has been described^48^ (Fig. 2d). Nonetheless, cell outlining from these images remains far from faithful (Fig. 2e,f). We decided that to reach the most precise localization, the cell outlines should be drawn from images with comparable resolution.

**Figure 3:**
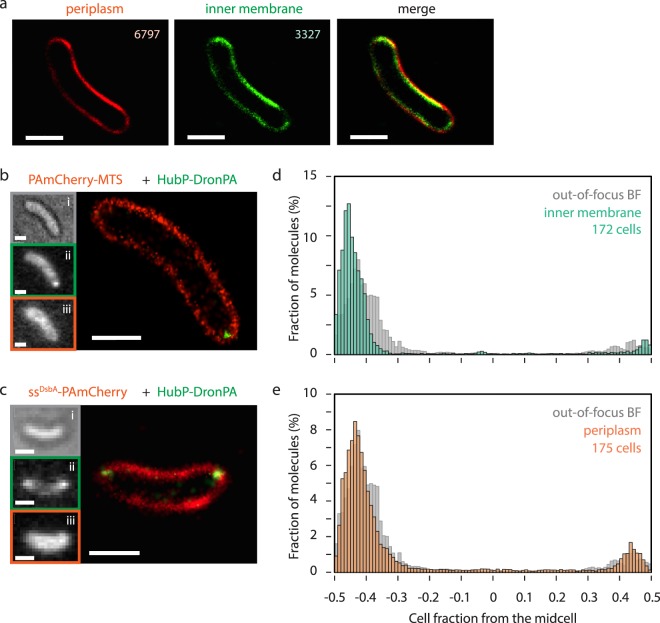
Novel cell outlining with photo-activatable/switchable fluorephores. (**a**) PALM of *V. cholerae* cells simultaneously expressing ss^DsbA^-PAmCherry and DronPA-MTS. Representative images reconstructed by N-STORM are shown. (**b**,**c**) Representative Images of HubP-DronPA (shown in green) with PAmCherry-MTS (**b**) or ss^DsbA^-PAmCherry (**c**) (shown in red). Out-of-focus BF image (i), conventional fluorescent images of HubP (ii), and outlining molecule (iii) are also shown. (**d**,**e**) Localization of HubP molecules relative to the cell length determined by the indicated outlining method. For better comparison, BF outlining results are recapitulated from Fig. 2e. Bar = 1 µm.

For that reason, we first tried to label the outer membrane of bacterial cells with PALM–compatible fluorescent dye, such as DiD cell labelling (ThermoFisher). However, it was aborted because it required fixation of cells which not only precluded live-cell imaging but also interfered with HubP-DronPA signals. Therefore we sought a protein-based approach to label either the inner cell membrane or the periplasm. For the former, we used short (21 amino acids) polypeptide from *B. subtilis* MinD (V199-S219) which is called membrane targeting sequence or MTS^49,50^, fused to DronPA or PAmCherry. For the later case, signal peptide of *E. coli* DsbA (ss^DsbA^) is fused to PAmCherry. In a proof-of-principle study, we constructed a plasmid (pEYY235) which encodes both *ss*^*DsbA*^*-PAmCherry* and *dronPA-MTS* under an arabinose-inducible promoter and introduced it in *V. cholerae*. With PALM, thousands of molecules of (ss^DsbA^-)PAmCherry and DronPA-MTS were detected. Importantly, they encompassed the entire cell contour in almost all of the cells, and periplasmic PAmCherry signals were found in the periphery of DronPA-MTS signals (Fig. 3a and Supplementary Fig. S2). Analysing intensities of reconstituted images confirmed that signals for periplasmic PAmCherry are slightly more external than those for inner membrane DronPA-MTS, and the difference can be as small as 30 nm (Supplementary Fig. S2) which correlates well with the width of the periplasmic space in the literature^51^. Re-analysing HubP-DronPA localization with this MTS labelling shifted the vast majority of HubP molecules to very close (<10%) to the cell pole (Fig. 3d), strongly indicating that this is a valid approach. As discussed above, HubP-DronPA localization in cells co-expressing ss^DsbA^-PAmCherry (thus outlined by the periplasm) showed less polar results compared to the inner membrane outlining (Fig. 3e and Supplementary Fig. S2c). Expression of these labelling markers did not cause any significant adverse effects on cell growth or morphology (Supplementary Fig. S1). Altogether, we developed a novel method suitable for PALM. Detailed operations to determine cell outline from such PALM images are shown in supplementary Fig. S3.

### Fine-scale quantitative analyses of polar localization in vibrioid cells

**Figure 4:**
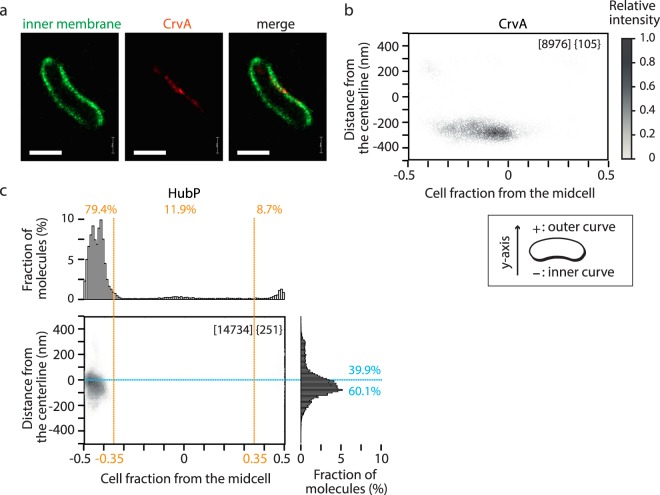
Fine-scale localization analyses by Vibio. (**a**) Representative processed PALM images of *V. cholerae* cell expressing DronPA-MTS (green) and CrvA-PAmCherry (red). Bar = 1 µm. (**b**) 2D plot presentation of CrvA-PAmCherry molecule localization. Cell outline was determined by DronPA-MTS. (**c**) Precise subcellular localization of HubP-DronPA molecules. Cell outline was determined by PAmCherry-MTS. 2D plot and histograms are shown. Percentage of molecules localized in specific fractions (polar, middle cell body, outer side, inner side) are indicated. Total number of molecules detected and number of cells analyzed are shown in square brackets and in braces, respectively.

*Vibrio* species are usually in curved rod shape and such ‘vibrioid’ morphology must have selective advantage in their life style^52^. Skewed protein localization between inner and outer curves in *V. cholerae* cells was recently shown with CrvA^32^. An oval fitting process in the Vibio pipeline, by definition, orients curved *V. cholerae* cells with respect to outer and inner curve. To confirm that Vibio can distinguish lopsided localization between inner and outer curves, we constructed a *V. cholerae* strain encoding PALM-compatible FP fusion of CrvA at the native locus (*crvA::crvA-PAmCherry*) and analysed the molecular distribution (Fig. 4a,b). The inner curve localization of CrvA was clearly presented in 2D plot (Fig. 4b), proving that Vibio is also a useful tool to analyse super-resolution microscopy data of vibrioid bacteria. Interestingly, when the distribution of HubP-DronPA molecules was analysed in 2D plot, it became apparent that the polar localization is in fact skewed towards the inner curve (Fig. 4c).

**Figure 5:**
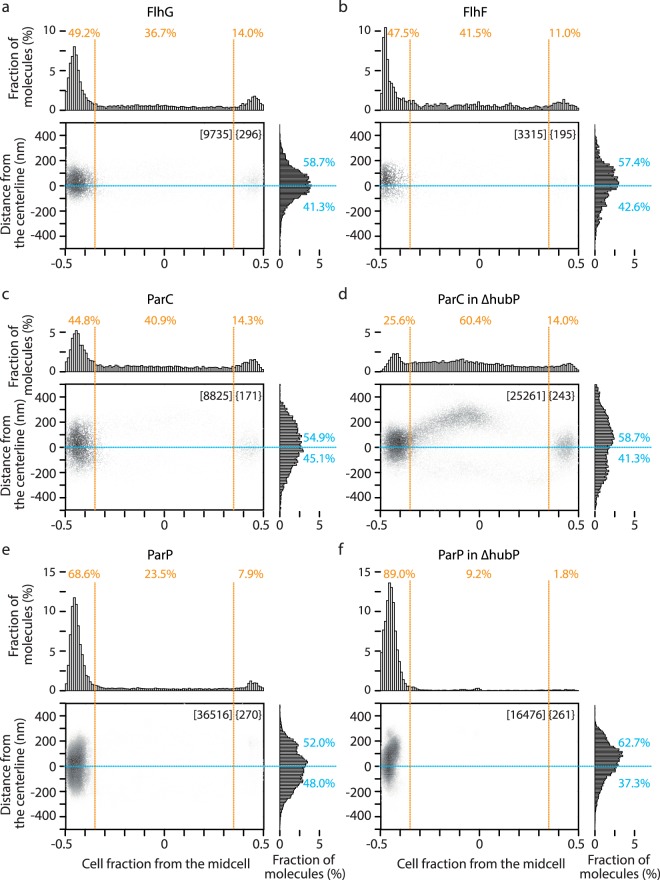
Fine-scale localization analyses of polar proteins. 2D plot and histogram presentations of DronPA fusions of FlhG (**a**), FlhF (**b**), ParC (**c**,**d**) and ParP (**e**,**f**) in *hubP*^+^ (**a**–**c**,**e**) or ∆*hubP* (**d**,**f**) background. PAmCherry-MTS was co-expressed for the determination of cell contour. Results are presented as described in Fig. 4c.

Next, we investigated the localization of other polar proteins including some interaction partners of HubP. Among 3 known HubP interaction partners (ParA1, ParC and FlhG), chromosomal FP fusion of ParA1 did not show fluorescence for unknown reasons. The fine-scale distribution of FlhG-DronPA and DronPA-ParC are indicated in Fig. 5a,c, respectively. Although they show uni- or bi-polar localization, considerable amounts of molecules were detected in the middle cell body, suggesting dynamic exchange of molecules from a cytoplasmic pool rather than fixed attachment. FlhG molecules were mainly found at the cell pole with a slight preference for the outer curve side. In contrast, ParC localization was more widely distributed at the cell pole, consistent with previous observations in conventional fluorescence microscopy^22^. While FlhG became diffuse in ∆*hubP* cells, ParC was shown to remain polar in ∆*hubP*, although exhibiting additional foci at non-polar region^22^. Analysis with super-resolution microscopy revealed that non-polar ParC showed strong preference for the outer curve over the inner curve (Fig. 5d).

FlhF is a positive regulator of flagella and conserved among many bacterial species^53^ and shown to interact with both FlhG and HubP, although the polar localization of FlhF is independent of HubP^22,24^. The molecular distribution of FlhF was similar to FlhG (Fig. 5b). Similar to FlhG-FlhF, the novel protein ParP was recently identified as a partner of ParC^54^. ParC and ParP regulate the localization of chemotaxis arrays by interaction to chemotaxis proteins CheW1 and CheA, respectively^54,55^. ParP was shown to be recruited to the cell pole by ParC but interaction with HubP was not discussed^54^. ParP showed a similar localization pattern to ParC with a wider distribution regarding the distance from centreline and closer to the cell pole (Fig. 5e). Unexpectedly, ParP showed a polar localization even in ∆*hubP* cells, but the distribution was visibly skewed to the outer curvature side (Fig. 5f). It is possible that ParP interaction to polar chemoreceptor arrays^54,55^ tethered it to the pole in *∆hubP* cells.

Altogether, while confirming polar localization, these proteins showed minor differences in fine-scale distribution such as fraction of non-polar, single molecules and detailed positioning towards the tip of the cell pole and the outer/inner curve.

## Discussion

In this study we used super-resolution PALM to address the precise localization of single molecules of the polar organizer protein HubP using DronPA and PAmCherry fusions. Unlike DronPA, PamCherry has appreciable tendency for oligomerization when fused to certain proteins (such as *E. coli* ClpP)^56,57^. However, HubP-DronPA and HubP-PAmCherry exhibited comparable subcellular localization at the cell poles. Therefore, HubP is unlikely to be a protein that causes unfavourable oligomerization of FP. Alternatively, it is also possible that HubP exists as an oligomer. Differences in uni-/bi- polarity of HubP by its expression level and the stage of the cell cycle^23^ were further confirmed by quantifying the number of molecules of HubP detected at each cell pole. In addition, we showed that HubP did not make a particular structure at the cell pole at a native expression level. When overexpressed, larger number of HubP molecules could turn into a cap-shaped structure. Yet, cluster analysis using SR-Tesseler indicated that HubP molecules are organized into only a single cluster at a cell pole in nearly all the cases regardless of overexpression or native expression level. Fluorescence recovery after photobleaching experiments suggested a dynamic exchange of HubP molecules between the cell pole and (membrane) pool^22^. We hypothesize that there is a certain capacity of HubP molecules at a cell pole, given accumulation of HubP molecules (presumably synthesized throughout the cell cycle), for transition from uni- to bi-polarity of HubP. The cap-shaped structure is likely an ‘overflow’ of overexpressed HubP molecules.

The determination of the exact cell body is critical for precise subcellular localization analysis. However, BF images combined to PALM did not give images of sufficient quality to draw accurate cell outlines in high-throughput analytic pipelines such as MicrobeTracker. One solution, implemented in HTPALM, relied on the usually incompatible phase contrast microscopy method instead of BF^46^. However, the localization analyses of polar proteins will be more affected by inaccurate outlining compared to the midcell localization of FtsZ^46^. Lastly, it is desirable to use comparable resolution to draw the cell outline. To this end, we developed a new method to outline either periplasm or inner membrane. Co-expression of ssDsbA-PAmCherry and DronPA-MTS strongly indicated that periplasm and inner membrane can be distinguished (Fig. 3a and Supplementary Fig. S2). Therefore, here we provide means to create accurate cell outlines for super-resolution analyses of both cytoplasmic and periplasmic proteins.

The distribution of molecules along the long axis showed differences in bi-polarity: while ~9% of the molecules were localized at the secondary (new) pole, ~14% of FlhG and ParC were found in the same region. It is known that HubP and its interaction partners show distinct patterns of uni- and bi- polarity, suggesting a certain ‘maturation of cell pole’ or ‘licensing’ process. It is puzzling why more interaction partners are distributed while a limited number of polar anchor is available at the new pole. A similar chicken-and-egg conundrum has been shown in the polar anchor and interaction partner in other bacteria. In *C. crescentus*, ZitP was identified as an interaction partner of PopZ, but overexpression of ZitP can modulate uni- to bi-polarity of PopZ^18^.

Fine-scale localization analysis with the high-throughput Vibio pipeline, which arranges cells with not only an old-new cell pole axis but also an inner-outer curve, unveiled skewed localization with respect to inner and outer curve of the cell. It is quite intriguing to elucidate the molecular mechanism(s) underneath the specific localization. With our precisely outlined and oriented analyses, the montage of 2D plots can provide some insights. However, a localization analysis of HubP and its partner protein in the same cell is legitimately required. On the contrary, colocalization in fluorescence microscopy images does not endorse physical apposition of two molecules^58^. Instead, it can be adequately appreciated for codistribution and molecular organization. For single molecule localization microscopy, distance-based methods have been developed to evaluate the spatial association, or coupling. Statistical Object Distance Analysis (SODA)^59^ showed that the vast majority of HubP molecules are coupled with other polar protein molecules, suggesting indirect interaction inside a macromolecular complex (distance less than 100 nm) (Supplementary Fig. S4). However, this analysis lacked information relative to the cell pole; since we could only use 2 colours in our PALM experiments, we were not able to draw precise cell outlines when two proteins were labelled with FPs.

Overall, our novel outlining technique and Vibio pipeline allows analysis of precise subcellular localization of molecules from dozens of super-resolution microscopy images and hundreds of cells while taking into account cell orientation correctly. Besides our application in polar proteins, these methods could provide better landscapes of the subcellular organization of other cellular machineries in small bacterial cells.

## Methods

### Bacterial strains, plasmids and media

Cholera toxin mutant (*∆ctx::kan*) of the wild type El Tor biovar *V. cholerae* N16961 was used in this study. Plasmids were constructed by either conventional digestion-ligation method or isothermal assembly^60^. Oligonucleotides coding the membrane target sequence (MTS) were generated by DNA polymerase reaction with overlapping primers as template. Signal sequence from *E. coli* DsbA (ss^DsbA^) was kindly provided by Dr. Thomas Bernhardt. Detailed constructions of plasmids are written in the supplementary text. Chromosomal insertions to express fluorescent protein fusions were done by allelic exchange. Resulting strains exhibited cell size, morphology, growth rate and motility comparable to the parental strain (Supplementary Fig. S1). Plasmids are introduced to *V. cholerae* by electroporation. Lists of plasmids, bacterial strains, and oligo nucleotides are indicated in Supplementary Tables S1–S3, respectively.

Unless specified, bacterial cells are grown in LB broth or agar (1.5%) and antibiotics are used at the following concentrations when appropriate: Ampicillin 100 µg/mL, Chloramphenicol 25 µg/mL (for *E. coli*) or 5 µg/mL (for *V. cholerae*), Kanamycin 25 µg/mL, Streptomycin 100 µg/mL.

Cell growth rate was measured by microplate reader (Tecan Infinite M200 Pro, Tecan Group, Switzerland)^61^. Cell motility/chemotaxis in soft (0.3%) agar plates was examined as previously described^61^.

### PALM imaging

Cells were grown in the M9 minimal media supplemented with glucose (0.2%), casamino acids (0.1%) and thiamine (1 µg/mL) at 37 °C with agitation (170 rpm). When necessary, fluorescent protein fusions encoded on overexpression plasmid were induced with 0.02% arabinose for 1 hour except HubP-PAmCherry which was induced with 0.2% arabinose for 4 hours.

Cover slips (thickness No. 1.5H) were pre-cleaned with acetone then plasma-cleaned for 10 min at 40 W with the ELMO glow discharge system (Cordouan Technology). Agarose pad (1% in 1x M9 media) was mounted on the slide glass using Gene Frame (Thermo Fisher). Far-red FluoSpheres beads (0.2 µm, Thermo Fisher) were pretreated by dilution with sterile purified H_2_O (1:1000) followed by sonication for 5 min. 1 µL of cell culture and 1 µL of beads solution were spread on the agarose pad then covered by the plasma-cleaned cover slip for microscopy.

Imaging was performed at the Imagif facility (Gif-sur-Yvette, France). All images were acquired with Nikon-Stochastic Optical Reconstruction Microscopy (N-STORM) at room temperature. The N-STORM was equipped with a CFI Apo TIRF SR 100 x oil immersion objective (NA 1.49), Coherent lasers emitting at 405 nm (100 mW), 488 nm (150 mW), 561 nm (150 mW) and built-in Nikon Perfect Focus system. Raw data were taken in a field of 256 × 256 pixels (40960 nm x 40960 nm) with an Andor iXon Ultra DU897 EM-CCD camera at a rate of 55 frames per second. The camera and microscope were controlled with the NIS-Elements Advanced Research software (version 5.01.00). Transmission z-stack containing 32 BF images covering positions from 1.6 µm below to 1.6 µm above the focal plane were performed before and after the molecule detection. Sequential acquisition option (one frame activation with 405 nm laser and one imaging with 488/561 nm lasers, sequentially) used for molecule detection during 2500–5000 frames. The overlapping peaks option was used to detect molecules that were in close proximity. Drift correction feature in NIS-Elements was applied to image analysis and the molecule list of each colour was exported in two different text files.

### Image analyses

For cluster analysis, SR-Tesseler^47^ which uses Voronoi diagram to subdivide a reconstructed PALM image into polygonal regions based on the distance between neighbour molecules, was used. The results were plotted with GraphPad Prism 7.0c.

To determine cell outlines with photo-activatable fluorophores, the molecule list obtained from N-STORM was converted to.csv format by using Fiji plugin ChriStorm^62^ and then loaded into ThunderSTORM^63^, which re-built a localization image in 1280 × 1280 pixels (40960 nm × 40960 nm) resolution. Bacterial cell shape was reconstructed then by using binary function on Fiji. The cell shape was drawn for all the outlining’s by MicrobeTracker^37^ version 0.937. Alternatively, conventional methods such as MicrobeTracker with BF images or contour detection script with z-stack of BF images^48^ were also used. Molecule list of the protein of interest and the cell outline information were subsequently combined by Vibio for further analysis.

Vibio is a Matlab based software with a graphical user interface. It was confirmed to run with Matlab R2015b and R2012b versions. The code is available in supplementary information (zip). Due to the death of CJM, however, it remained incomplete: e.g. the output of multifield data is not automatic but requires the manual integration of single-field data (which contains data from multiple cells). Combined data were visualized with Matlab (histograms) and Wolfram Mathematica 11 (scatter density plots).

The spatial coupling between two fluorescent molecules (Supplementary Fig. S4) was investigated by using the SODA 2D-STORM plug-in^59^ in Icy. Briefly, dual color PALM data were exported into two files depending on the labelling (channel). 7–10 PALM images for each construction were analysed to calculate the number of isolated and coupled molecules as well as the average distance.

To examine cell size and morphology of different strains, cells were grown in the M9 minimal media as for PALM imaging and 1 µL of culture was spotted on the agarose pad. Phase contrast images were taken with a Zeiss axio observer Z1 microscope, and Evolve EM-CDD camera (Roper) and Axio vision software. Image analysis was performed with MicrobeTracker.

### Gene expression analyses

Cells were grown in the M9 minimal media as for PALM imaging. Monarch Total RNA miniprep kit (New England Biolabs) was used to purify RNA from enzymatically lysed cells according to the manufacture’s instructions. 2 ng of total RNA was used for One-Step RT-qPCR (New England Biolabs) with LightCycler 480 (Roche) to quantify transcription level for *hubP*. A housekeeping gene *rpoB* was used for the control. Nucleotide sequence of primers (oYo824-825 and oYo822-823 for *hubP* and *rpoB*, respectively) is provided in Supplementary Table S3.

## Supplementary information

supplementary information

Supplementary Dataset 2

## Data Availability

The datasets generated during the current study are available from the corresponding author on reasonable request.
