# Supplementary material for: Single molecule super-resolution imaging of bacterial cell pole proteins with high-throughput quantitative analysis pipeline
Source: Sci Rep. 2019 Apr 30;9:6680. doi: 10.1038/s41598-019-43051-7 (PMC6491441; doi:10.1038/s41598-019-43051-7)
Supplement: Supplementary file 1 — supplementary information [file 41598_2019_43051_MOESM1_ESM.pdf]

SUPPLEMENTARY INFORMATION

Single molecule super-resolution imaging of bacterial cell pole proteins with high-throughput quantitative analysis pipeline

Ipek Altinoglu [1,3], Christien J. Merrifield [2†], Yoshiharu Yamaichi [1*]

[1] Department of Genome Biology, Institute for Integrative Biology of the Cell (I2BC), Université Paris-Saclay, CEA, CNRS, Univ. Paris Sud, Gif sur Yvette, FRANCE

[2] Department of Cell Biology, Institute for Integrative Biology of the Cell (I2BC), Université Paris-Saclay, CEA, CNRS, Univ. Paris Sud, Gif sur Yvette, FRANCE

[3] Graduate School of Structure and Dynamics of Living Systems, Univ. Paris-Sud, Orsay, FRANCE

† Deceased

Supplementary Methods

- **Supplementary Table S1 : Plasmids used in this study**
- **Supplementary Table S2 : Strains used in this study**
- **Supplementary Table S3 : Oligonucleotides used in this study**
- **Legend to Supplementary Figures**
- **References for Supplementary Information**
- **Supplementary Figure S1: No adverse affects of fluorescent protein fusions**
- **Supplementary Figure S2: Specificity of novel cell outlining**
- **Supplementary Figure S3: Schematics of outlining methods**
- **Supplementary Figure S4: Molecular coupling of HubP and partner proteins**

Script of Vibio (in separated file)

Supplementary Methods

Construction of gene expression plasmids

pEYY99, pEYY133 and pEYY134 were designed to simplify subsequent constructions of plasmids for the expression of fusion proteins. *dronPA* (for pEYY99) and *PAmCherry* (for pEYY133 and pEYY134) were amplified with oYo315 and oYo316, oYo352 and oYo353, oYo350 and oYo351, respectively then cloned into the SacI-XbaI sites of pBAD33.

hubP gene was excised from pEYY470 {Yamaichi 2012} by NotI digestion and ligated into pEYY99 and pEYY133, resulting in pEYY109 and pEYY135, respectively. Other genes (*flhF*, *flhG*) and the signal sequence (ss^{DsbA}) were amplified with oYo562 and oYo563, oYo564 and oYo565, pBAD_F and oYo493, respectively, followed by insertion to the vector by isothermal assembly (Gibson et al., 2009).

To construct PAmCherry-MTS fusion plasmid, two overlapping oligonucleotides oYo356 and oYo357 were annealed then treated with Taq polymerase. Resulting double-strand DNA was digested with BsrGI-SalI and ligated into the corresponding sites of pEYY134. *PAmCherry* gene was excised by SacI-BsrGI digestion then replaced by *dronPA* (amplified with oYo412 and oYo413), resulting in *dronPA-MTS* fusion plasmid pEYY166.

To construct composite expression plasmids, pEYY235 was constructed by ligation of *dronPA-MTS*, which was amplified with oYo577 and pBAD_R followed by digestion with XhoI-HindIII, into SalI-HindIII sites of pEYY225. For pEYY304, *PAmCherry-MTS* was excised from pEYY137 by NheI-SalI digestion then ligated into XbaI-SalI sites of pEYY109.

Construction of allelic exchange plasmids

For chromosomal *hubP* fusions, *yfp* gene of pYB517 was replaced by *dronPA* or *PAmCherry* that were amplified with oYo93 and oYo94 or oYo354 and oYo355, respectively.

Plasmids for *flhF-dronPA* and *flhG-dronPA* chromosomal fusions were constructed by isothermal assembly of 2+1 (fluorescent protein fusion gene, downstream region + vector) fragments. pCVD442 vector was linearized by SmaI digestion. Oligonucleotides used for amplification were as following: pEYY285; oYo689 and oYo691 + oYo690 and oYo692, pEYY305; oYo248 and oYo687 + oYo243 and oYo688.

Other plasmids for chromosomal fusions were constructed by isothermal assembly of 3+1 (upstream region, fluorescent protein gene, downstream region + vector) fragments. pCVD442 vector was linearized by SmaI digestion. Oligonucleotides used for amplification were as following: pEYY202; oYo496 and oYo498 + oYo499 and oYo501 + oYo497 x oYo500, pEYY214; oYo521 and oYo523 + oYo524 and oYo526 + oYo522 and oYo525, pEYY284; oYo693 and oYo695 + oYo696 and oYo698 + oYo694 and oYo697.

Supplementary Table S1 : Plasmids used in this study

Plasmid	Description	Reference
Gene expression plasmids		
pBAD33	vector; P15A*ori*, P_{ara}, CmR	(Guzman et al. 1995)
pEYY99	cloning vector for C-terminal DronPA fusion	This study
pEYY133	cloning vector for C-terminal PAmCherry fusion	This study
pEYY134	cloning vector for N-terminal PAmCherry fusion	This study
pYB470	pBAD33 *hubP-yfp*	(Yamaichi et al. 2012)
pEYY109	pBAD33 *hubP-dronPA*	This study
pEYY135	pBAD33 *hubP-PAmCherry*	This study
pEYY137	pBAD33 *PAmCherry-MTS*	This study
pEYY166	pBAD33 *dronPA-MTS*	This study
pEYY225	pBAD33 ss^{DsbA}*-PAmCherry*	This study
pEYY228	pBAD33 *flhF-dronPA*	This study
pEYY229	pBAD33 *flhG-dronPA*	This study
pEYY235	pBAD33 ss^{DsbA}*-PAmCherry dronPA-MTS*	This study
pEYY304	pBAD33 *hubP-dronPA PAmCherry-MTS*	This study
Allelic exchange plasmids		
pCVD442	cloning vector; R6K*ori*, AmpR, sucB	(Donnenberg & Kaper 1991)
pYB517	for *hubP::hubP-yfp*	(Yamaichi et al. 2012)
pEYY30	for *hubP::hubP-dronPA*	This study
pEYY140	for *hubP::hubP-PAmCherry*	This study
pEYY202	for *parC::dronPA-parC*	This study
pEYY214	for *crvA::crvA-PAmCherry*	This study
pEYY284	for *parP::PAmCherry-parP*	This study
pEYY285	for *flhF::flhF-dronPA*	This study
pEYY305	for *flhG::flhG-dronPA*	This study

Supplementary Table S2 : Strains used in this study

Strain	Description	Reference
Escherichia coli		
DH5α	host strain of general cloning	laboratory strain
DH5α λ*pir*	host strain for the cloning of R6K*ori* plasmid	laboratory strain
SM10 λ*pir*	host strain for conjugation	laboratory strain
Vibrio cholerae		
N16961	*V. cholerae* O1 El Tor, SmR	(Heiderberg et al. 2000)
MKW1383	N16961 Δ*ctxAB::kan*	M. Waldor
bEYY1014	N16961 Δ*ctxAB::kan* Δ*hubP*	this study
bEYY1068	N16961 Δ*ctxAB::kan hubP::hubP-dronPA*	this study
bEYY1274	N16961 Δ*ctxAB::kan* / pEYY109	this study
bEYY1345	N16961 Δ*ctxAB::kan* / pEYY135	this study
bEYY1354	N16961 Δ*ctxAB::kan* / pEYY137	this study
bEYY1355	N16961 Δ*ctxAB::kan hubP::hubP-dronPA* / pEYY137	this study
bEYY1356	N16961 Δ*ctxAB::kan hubP::hubP-PAmCherry*	this study
bEYY1360	N16961 Δ*ctxAB::kan* / pBAD33	this study
bEYY1445	N16961 Δ*ctxAB::kan* / pEYY166	this study
bEYY1448	N16961 Δ*ctxAB::kan hubP::hubP-PAmCherry* / pEYY166	this study
bEYY1634	N16961 Δ*ctxAB::kan parC::dronPA-parC*	this study
bEYY1660	N16961 Δ*ctxAB::kan hubP::hubP-PAmCherry parC::dronPA-ParC*	this study
bEYY1661	N16961 Δ*ctxAB::kan crvA::crvA-PAmCherry*	this study
bEYY1662	N16961 Δ*ctxAB::kan hubP::hubP-dronPA crvA::crvA-PAmCherry*	this study
bEYY1690	N16961 Δ*ctxAB::kan* / pEYY225	this study
bEYY1697	N16961 Δ*ctxAB::kan hubP::hubP-dronPA* / pEYY225	this study
bEYY1712	N16961 Δ*ctxAB::kan* / pEYY235	this study
bEYY1721	N16961 Δ*ctxAB::kan crvA::crvA-PAmCherry* / pEYY166	this study
bEYY1731	N16961 Δ*ctxAB::kan parC::dronPA-ParC* / pEYY137	this study
bEYY1770	N16961 Δ*ctxAB::kan* Δ*hubP parC::dronPA-parC*	this study
bEYY1771	N16961 Δ*ctxAB::kan* Δ*hubP parC::dronPA-parC*	this study
bEYY1781	N16961 Δ*ctxAB::kan* Δ*hubP parC::dronPA-parC* / pEYY137	this study
bEYY1826	N16961 Δ*ctxAB::kan hubP::hubP-PAmCherry flhF::flhF-dronPA*	this study
bEYY1839	N16961 Δ*ctxAB::kan parP::PAmCherry-parP*	this study
bEYY1840	N16961 Δ*ctxAB::kan* Δ*hubP parP::PAmCherry-parP*	this study
bEYY1841	N16961 Δ*ctxAB::kan hubP::hubP-dronPA parP::PAmCherry-parP*	this study
bEYY1842	N16961 Δ*ctxAB::kan parC::dronPA-parC parP::PAmCherry-parP*	this study
bEYY1843	N16961 Δ*ctxAB::kan* Δ*hubP flhF::flhF-dronPA*	this study
bEYY1844	N16961 Δ*ctxAB::kan hubP::hubP-PAmCherry flhG::flhG-dronPA*	this study
bEYY1845	N16961 Δ*ctxAB::kan* Δ*hubP flhG::flhG-dronPA*	this study
bEYY1846	N16961 Δ*ctxAB::kan flhG::flhG-dronPA*	this study
bEYY1847	N16961 Δ*ctxAB::kan flhF::flhF-dronPA*	this study
bEYY1854	N16961 Δ*ctxAB::kan* Δ*hubP parP::PAmCherry-parP* / pEYY166	this study
bEYY1855	N16961 Δ*ctxAB::kan flhG::flhG-dronPA* / pEYY137	this study
bEYY1856	N16961 Δ*ctxAB::kan* Δ*hubP flhG::flhG-dronPA* / pEYY137	this study
bEYY1857	N16961 Δ*ctxAB::kan* Δ*hubP flhF::flhF-dronPA* / pEYY137	this study
bEYY1858	N16961 Δ*ctxAB::kan parP::PAmCherry-parP* / pEYY166	this study
bEYY1858	N16961 Δ*ctxAB::kan parP::PAmCherry-parP* / pEYY166	this study
bEYY1859	N16961 Δ*ctxAB::kan flhF::flhF-dronPA* / pEYY137	this study
bEYY1879	N16961 Δ*ctxAB::kan* / pEYY304	this study
bEYY1886	N16961 Δ*ctxAB::kan hubP::hubP-PAmCherry* / pEYY109	this study
bEYY1858	N16961 Δ*ctxAB::kan parP::PAmCherry-parP* / pEYY166	this study

Supplementary Table S3 : Oligonucleotides used in this study

Name	Sequence (5'-3')
oYo93	GCTGAATGGGCGAGTGGGTGGTGCGGCCGCCGGTGGCAGTGTGATTAAACCAGACATG
oYo94	GTCGAAAGAATCAGAGGGAGGCTAGCTTACTTGGCCTGCCTCGGCAG
oYo243	CCGCATGCGATATCGAGCTCTCCCGCTCTCTCATGCTCAGCAC
oYo248	CGGATAACAATTTGTGGAATTCCCAAGTACCAAAGCTTCACGC
oYo315	AGCTGAGCTCAAGGAGGAGCGGCCGCCAGTGTGATTAAACCAGACATG
oYo316	GCTCTAGATTACTTGGCCTGCCTCGGCAG
oYo350	ATTCGAGCTCAAGGAGGAAAACCATGGTGAGCAAGGGCGAG
oYo351	GGTCGACTCTAGATTAGGCGGCCGCCCTTGTACAGCTCGTCCATGCC
oYo352	AGCTGAGCTCAAGGAGGAGCGGCCGCCGTGAGCAAGGGCGAGGAG
oYo353	GCTCTAGATTACTTGTACAGCTCGTCCATGC
oYo354	GCTGAATGGGCGAGTGGGTGGTGCGGCCGCCGTGAGCAAGGGCGAGGAG
oYo355	GTCGAAAGAATCAGAGGGAGGCTAGCTTACTTGTACAGCTCGTCCATGC
oYo356	AGCTGTACAAGGTGCTTGAAGAGCAAAACAAAGGAATGATGGCTAAGATTAAGTCATTTT
oYo357	GCAGGTCGACTCTAGTTAAGATCTTACTCCGAAAAATGACTTAATCTTAGCCATCATTCC
oYo412	GAATTCGAGCTCAAGGAGGAAAACCATGAGTGTGATTAAACCAGACATG
oYo413	CTTCAAGCACCTTGTACAACTTGGCCTGCCTCGGCAG
oYo493	GTTATCCTCCTCGCCCTTG
oYo496	CCGCATGCGATATCGAGCTCTCCCGCGATTTCTGCATAATTGCC
oYo497	GGTTTGCTACACTCCAAACGATGGCGGCCGCCTTGGCCTGCCTCGGCAGC
oYo498	GCTGCCGAGGCAGGCCAAGGCGGCCGCCATCGTTTGGAGTGTAGCAAACC
oYo499	GTCTGGTTTAATCACACTCATTGAGGTTCCTTAGGCGAGCCCGACTTC
oYo500	GAAGTCGGGCTCGCCTAAGGAACCTCAATGAGTGTGATTAAACCAGAC
oYo501	CGGATAACAATTTGTGGAATTCCCCCGTAAATTATCCACACCC
oYo521	CCGCATGCGATATCGAGCTCTCCCCGACTTGGTTCTGTTTGACC
oYo522	GAAATCGTTGGATTTTTGTGCGGTTACTTGTACAGCTCGTCCATGC
oYo523	GCATGGACGAGCTGTACAAGTAACCGCACAAAAATCCAACGATTTC
oYo524	CTCCTCGCCCTTGCTCACCACGGCGGCCGCGCTGTCTTTGTTTGGTCTG
oYo525	CAGACCAAACAAAGACAGCGCGGCCGCCGTGGTGAGCAAGGGCGAGGAG
oYo526	CGGATAACAATTTGTGGAATTCCCCAGGGCCAATACCTGACG
oYo562	TGGGCTAGCGAATTCGAGCTTTGAAAATAAAACGATTTTTTGC
oYo563	CATGTCTGGTTTAATCACACTGGCGGCCGCGAATCTCTCTGAATCACTG
oYo564	TGGGCTAGCGAATTCGAGCTATGAACCTCATTACTGGACC
oYo565	CATGTCTGGTTTAATCACACTGGCGGCCGCTTCACCGAAAGGTTCCC
oYo577	AATCCTCGAGGAGCTCAAGGAGGAAAACC
oYo687	CCGAGGCAGGCCAAGTAAGGAACCTTTCGGTGAATAAAGCGCTTACATACG
oYo688	CGTATGTAAGCGCTTTATTCACCGAAAGGTTCCTTACTTGGCCTGCCTCGG
oYo689	CGGATAACAATTTGTGGAATTCCCAGACACTTACCGTATTGGCGCCCATG
oYo690	GCCGAGGCAGGCCAAGTAAGTCGACTGAAGATGAACCTCATTACTGGACC
oYo691	GGTCCAGTAATGAGGTTCATCTTCAGTCGACTTACTTGGCCTGCCTCGGC
oYo692	CCGCATGCGATATCGAGCTCTCCCCCACCATATCTGAGATACCTGCCG
oYo693	CGGATAACAATTTGTGGAATTCCCGCGCCTCAACAACCGGTACC
oYo694	GCTGGATATCAATGCACTACTGGCGGCCGCCTTGTACAGCTCGTCCATGC
oYo695	GCATGGACGAGCTGTACAAGGCGGCCGCCAGTAGTGCATTGATATCCAGC
oYo696	CCTTGCTCACCATGGTTTTCCTCCTTGCTACTCATCGATAGCGAGCCTCTC
oYo697	GAGAGGCTCGCTATCGATGAGTAGCAAGGAGGAAAACCATGGTGAGCAAGG
oYo698	CCGCATGCGATATCGAGCTCTCCCCCCATATGTCTCTTGCGACACTGG
oYo822	TGGACAGTTTGGGTCACTTATC
oYo823	GGACGAGTACCAAAGTCCTTAC
oYo824	GATGAGAACCGCATCCG
oYo825	ATCTAAAGCTGATGGCGC
pBAD_F	ATGCCATAGCATTTTTATCC
pBAD_R	GATTTAATCTGTATCAGG

Legend to Supplementary Figures

SUPPLEMENTARY FIGURE S1. No adverse affects of fluorescent protein fusions. (a) Growth rate of indicated *V. cholerae* strains. Average and standard deviations of 3 independent experiments are shown. (b) Cell motility/chemotaxis measured by soft agar plate assay. Diameters relative to the control strain (wild type *ctx*$^-$ or wild type *ctx*$^-$ harboring pBAD33 vector) were measured in 4 independent experiments and the average and standard deviations are shown. (c) Transcription of *hubP*, standardized by *rpoB* expression. Average and standard deviations of 4 independent experiments are shown. (d) Distribution of cell length presented by box plot with 10th and 90th percentile whiskers. Total number of cells investigated are indicated in parentheses. Representative phase contrast cell images are also shown in (e). Bar = 2 µm

SUPPLEMENTARY FIGURE S2. Specificity of novel cell outlining. (a and b) Representative cells (other than the one shown in Fig. 3a) for duel color labelling by ss^{DsbA}-PAmCherry (red) and DronPA-MTS (green). Images are reconstructed by using the Gaussian Rendering Parameters in N-STORM. Number of molecules detected is shown in parentheses. The average and standard deviations for Lateral Localization Accuracy (LLA) are also shown. Bar = 500 nm. Four points (1~4, with the width of 128 subpixels) were chosen for intensity profile analysis shown on the right. Arrowheads represent the direction of measurement. (c) Box plot comparison of HubP molecule distribution. Localization of molecules was measured from the closest cell pole with different labelling techniques. Whiskers represent 10th and 90th percentiles. * $p<0.001$ for one-way ANOVA/Tukey's multiple comparison test.

SUPPLEMENTARY FIGURE S3. Schematics of outlining methods. (a) Representative images of Brightfield (BF) of -200 nm of focal plane (i) and subsequent outline (shown in green) determined by MicrobeTracker (ii). Bar = 3 µm. (b) Z-stack outlining by the method described in (Demarre et al., 2014). Compressed image of 32 z-stacks (i) and subsequent segmentation (ii) are shown. (c) Novel MTS-labeling with PALM. (i) Representative single image of 2500 PALM acquisitions of PAmCherry-MTS. Fluorescent beads for correlation of drifting are indicated by arrowheads. (ii) Compiled single molecule localization image is illustrated with red cross marking in N-STORM. Single molecule localizations in x,y-coordinates (iii) were exported to reconstruct image (iv) in 1280 x 1280 pixels (40960 nm x 40960 nm) resolution by ImageJ plug-in ThunderStorm. Binary function was used to illustrate phase contrast-like image (v). This image was subsequently treated with MicrobeTracker (vi) which gives cellList file in MatLab (vii).

SUPPLEMENTARY FIGURE S4. Molecular coupling of HubP and partner proteins. (a) Representative images of SODA analysis with HubP-PAmCherry and FlhG-DronPA coupling. Each molecule was classified coupled or uncoupled as indicated. (b) Results of coupling. Total number of detected molecules and fraction of coupled molecules for each FP fusion were shown along with Venn diagram (with Venn Diagram Plotter, http://omics.pnl.gov/software/venn-diagram-plotter).

References for Supplementary Materials

Demarre, G. *et al*. Differential Management of the Replication Terminus Regions of the Two *Vibrio cholerae* Chromosomes during Cell Division. *PLoS Genet* **10**, e1004557 (2014).

Donnenberg, M. S. & Kaper, J. B. Construction of an eae deletion mutant of enteropathogenic *Escherichia coli* by using a positive-selection suicide vector. *Infect Immun* **59,** 4310-4317 (1991).

Gibson, D. G. *et al*. Enzymatic assembly of DNA molecules up to several hundred kilobases. *Nat Methods* **6,** 343-345 (2009).

Guzman, L. M., Belin, D., Carson, M. J. & Beckwith, J. Tight regulation, modulation, and high-level expression by vectors containing the arabinose PBAD promoter. *J Bacteriol* **177,** 4121-4130 (1995).

Heidelberg, J. F. *et al*. DNA sequence of both chromosomes of the cholera pathogen *Vibrio cholerae*. *Nature* **406,** 477-483 (2000).

Yamaichi, Y. *et al*. A multidomain hub anchors the chromosome segregation and chemotactic machinery to the bacterial pole. *Genes Dev* **26,** 2348-2360 (2012).

Supplementary Figure S1

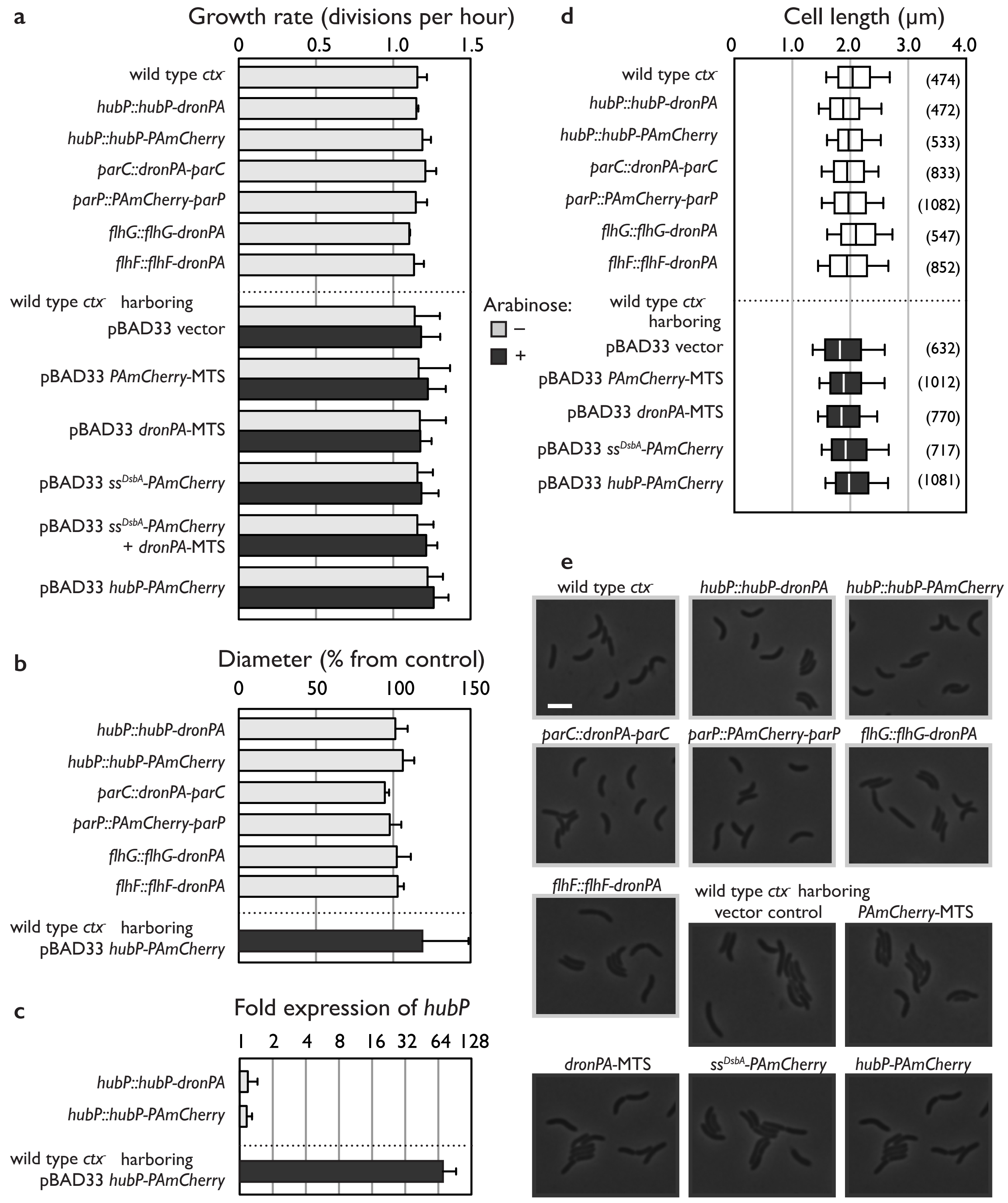

Supplementary Figure S1. No adverse affects of fluorescent protein fusions.
(a) Growth rate of indicated *V. cholerae* strains. Average and standard deviations of 3 independent experiments are shown. (b) Cell motility/chemotaxis measured by soft agar plate assay. Diameters relative to the control strain (wild type *ctx*- or wild type *ctx*- harboring pBAD33 vector) were measured in 4 independent experiments and the average and standard deviations are shown. (c) Transcription of *hubP*, standardized by *rpoB* expression. Average and standard deviations of 4 independent experiments are shown. (d) Distribution of cell length presented by box plot with 10th and 90th percentile whiskers. Total number of cells investigated are indicated in parentheses. Representative phase contrast cell images are also shown in (e). Bar = 2 µm

Supplementary Figure S2

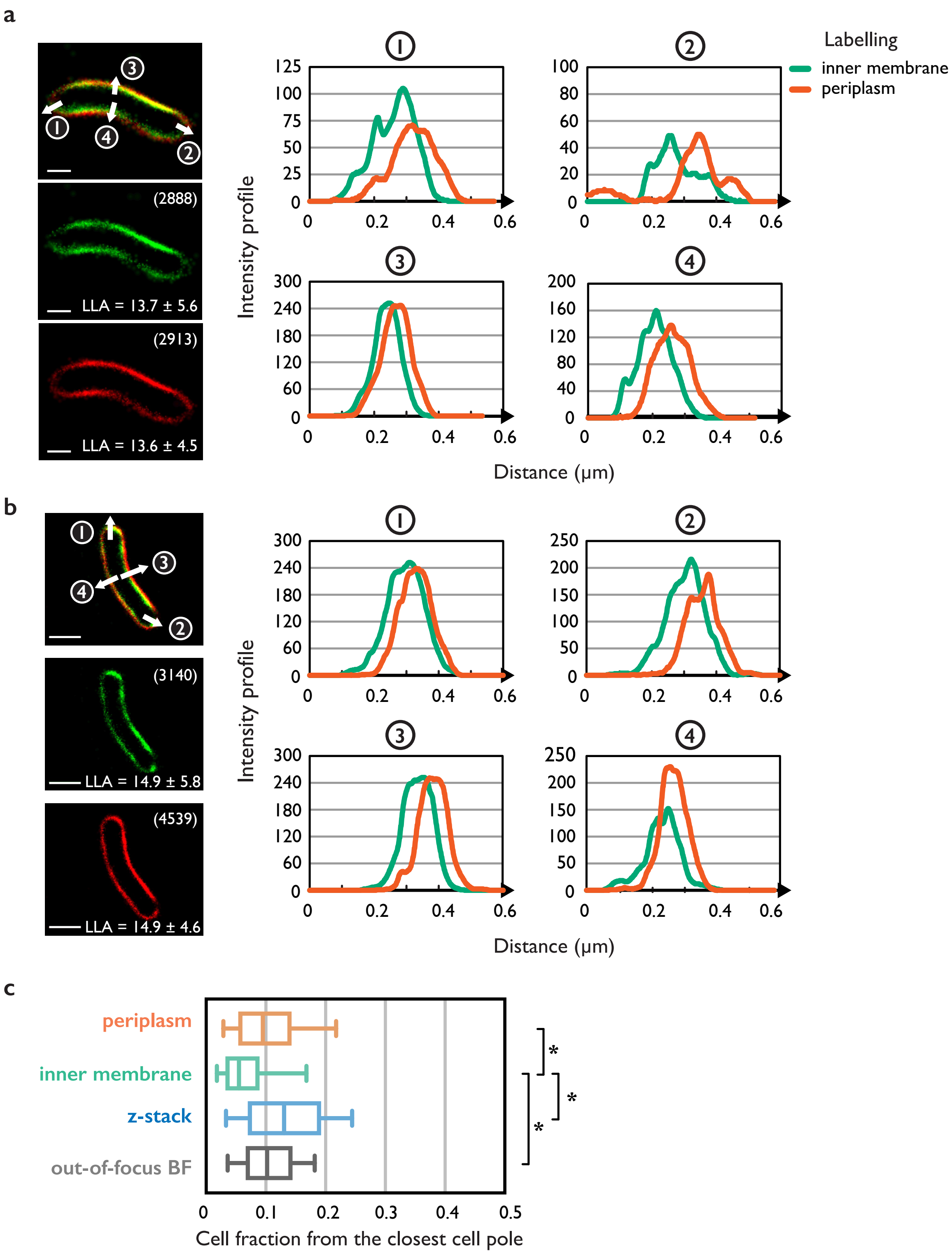

Supplementary Figure S2. Specificity of novel cell outlining. (a and b) Representative cells (other than the one shown in Fig. 3a) for duel color labelling by ssDsbA-PAmCherry (red) and DronPA-MTS (green). Images are reconstructed by using the Gaussian Rendering Parameters in N-STORM. Number of molecules detected is shown in parentheses. The average and standard deviations for Lateral Localization Accuracy (LLA) are also shown. Bar = 500 nm. Four points (1~4, with the width of 128 subpixels) were chosen for intensity profile analysis shown on the right. Arrowheads represent the direction of measurement. (c) Box plot comparison of HubP molecule distribution. Localization of molecules was measured from the closest cell pole with different labelling techniques. Whiskers represent 10th and 90th percentiles. * $p<0.001$ for one-way ANOVA/Tukey's multiple comparison test.

Supplementary Figure S3

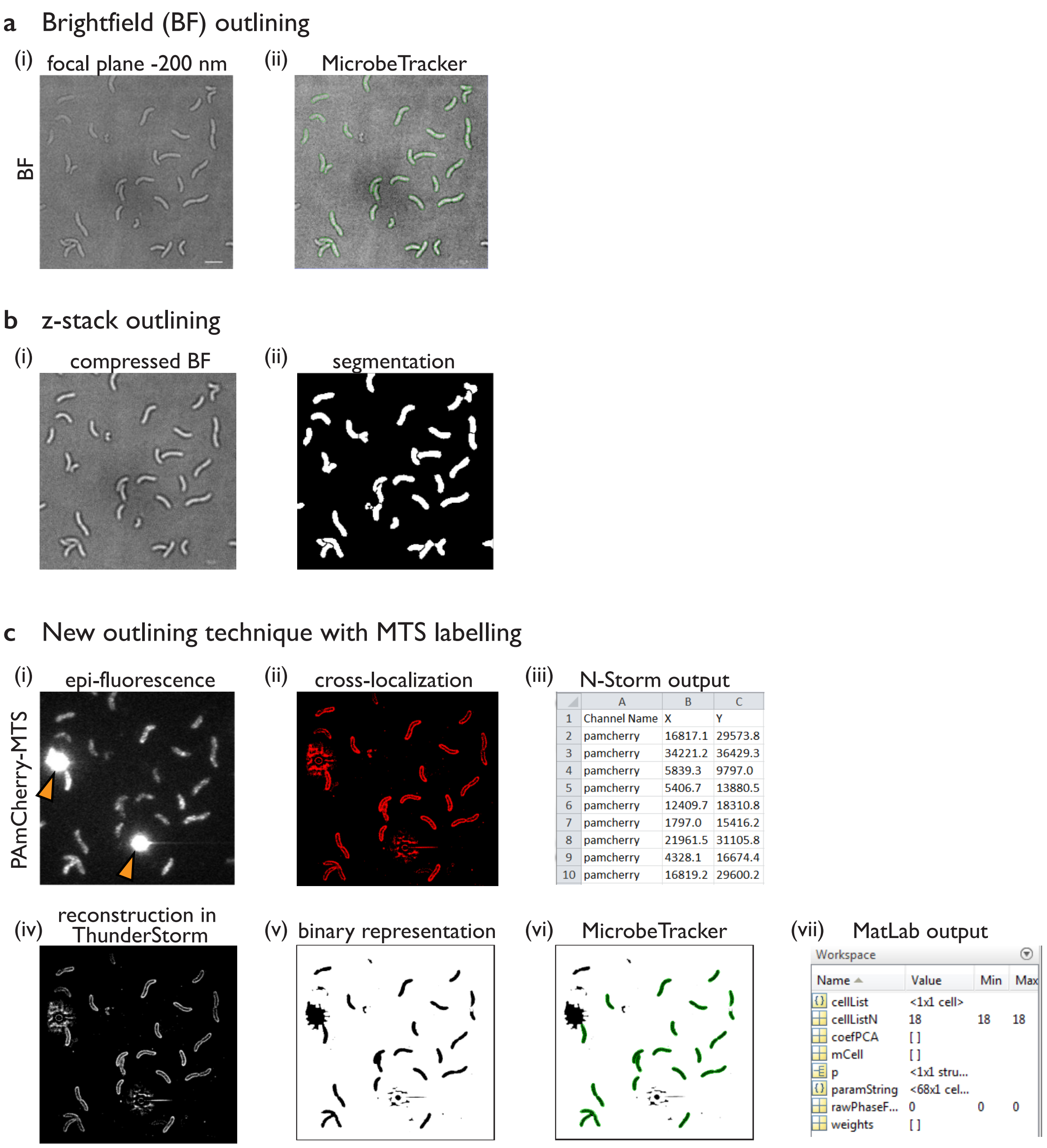

Supplementary Figure S3. Schematics of outlining methods.
(a) Representative images of Brightfield (BF) of -200 nm of focal plane (i) and subsequent outline (shown in green) determined by MicrobeTracker (ii). Bar = 3 μm. (b) Z-stack outlining by the method described in (Demarre et al., 2014). Compressed image of 32 z-stacks (i) and sebsequent segmentation (ii) are shown. (c) Novel MTS-labeling with PALM. (i) Representative single image of 2500 PALM acquisitions of PAmCherry-MTS. Fluorescent beads for correlation of drifting are indicated by arrowheads. (ii) Compiled single molecule localization image is illustrated with red cross marking in N-STORM. Single molecule localizations in x,y-coordinates (iii) were exported to reconstruct image (iv) in 1280 x 1280 pixels (40960 nm x 40960 nm) resolution by ImageJ plug-in ThunderStorm. Binary function was used to illustrate phase contrast-like image (v). This image was subsequently treated with MicrobeTracker (vi) which gives cellList file in MatLab (vii).

Supplementary Figure S4

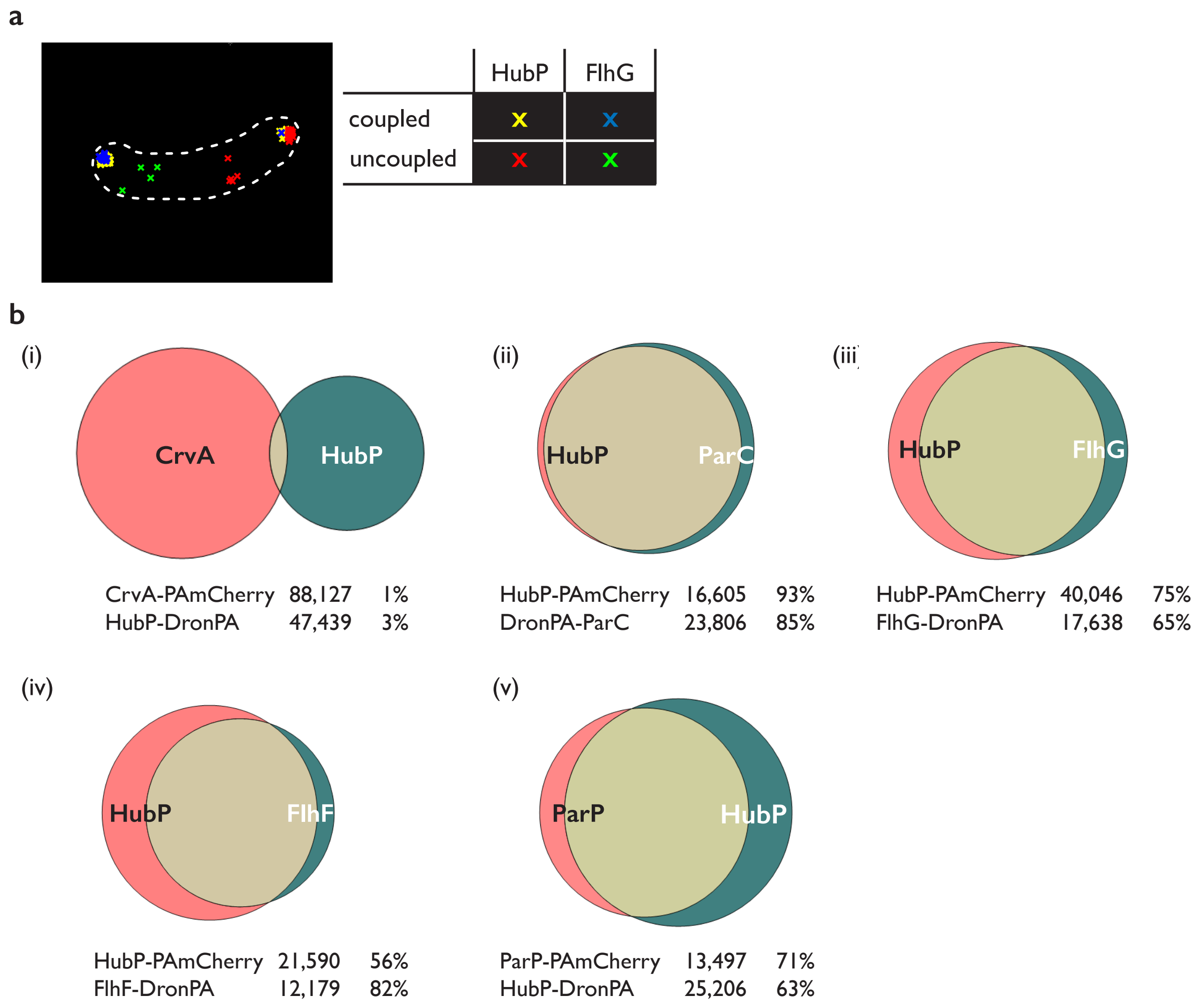

Supplementary Figure 4. Molecular coupling of HubP and partner proteins. (a) Representative images of SODA analysis with HubP-PAmCherry and FlhG-DronPA coupling. Each molecule was classified coupled or uncoupled as indicated. (b) Results of coupling. Total number of detected molecules and fraction of coupled molecules for each FP fusion were shown along with Venn diagram (with Venn Diagram Plotter, http://omics.pnl.gov/software/venn-diagram-plotter).
